# Understanding, detecting, and managing strabismus

**Published:** 2010-03

**Authors:** Eugene M Helveston

**Affiliations:** Director, ORBIS Telemedicine, Cyber-Sight, ORBIS International, 520 8th Ave, New York, NY 10018, USA.

**Figure:**
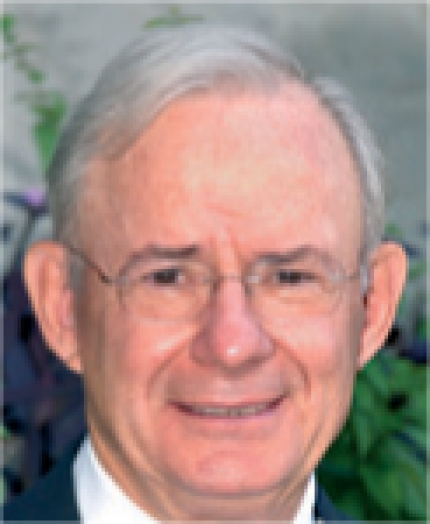


Whereas many animals have eyes located on either side of their head (such as horses, for example), the eyes of humans look forwards - in the same direction. When normal, the eyes move in a coordinated manner, so that the object being looked at is centred in each eye. Because the eyes are set a small distance apart, the image in each eye is slightly different. The brain fuses the images coming from both eyes to produce a three-dimensional image that has depth. This three-dimensional vision, also known as stereoscopic or binocular vision, gives us depth perception. This allows us to judge distances more accurately, especially with objects close to us. Try to thread a needle with only one eye open and you will see the advantage of binocular vision!

In order to achieve normal binocular vision, the eyes must see well, be aligned (looking in the same direction), and be focused properly on the same object. To maintain alignment, the eyes must also move in a coordinated manner, a process involving twelve different muscles (six in each eye). The four rectus muscles move the eyes up, down, to the right, and to the left, and the two oblique muscles have more complex actions, helping the eyes to look down and in (towards the tip of the nose) or up and in (towards the bridge of the nose). Three different cranial nerves are involved in the contraction and relaxation of these muscles and the main coordinating centre is in the brain.

Misalignment of the eyes is called **strabismus** (or **squint**). Misalignment means that the eyes are not lined up to look at the same thing. In every case of strabismus or misalignment, one eye is fixed on what the person intends to look at (the fixing eye) and the other eye is looking at something else (the deviated eye).

**Figure:**
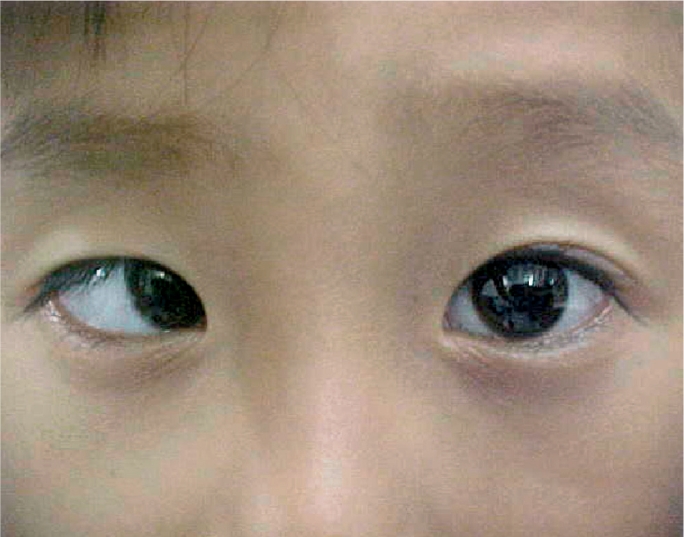
Figure 1. This boy's right eye is deviated inwards, an example of esotropia

ORBIS

**Figure:**
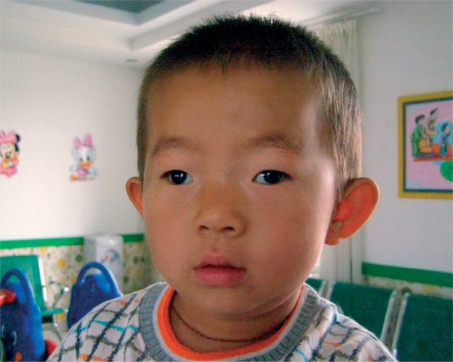
Figure 2. This boy's right eye is deviated outwards, an example of exotropia

ORBIS

**Figure:**
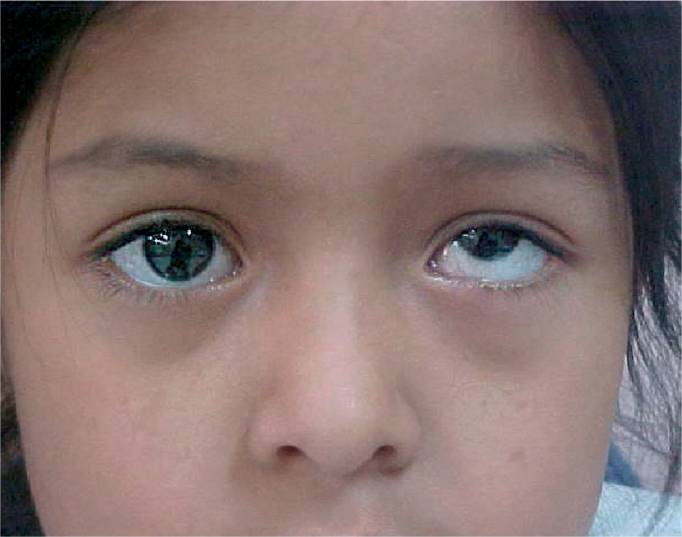
Figure 3. The left eye is higher in this girl, an example of hypertropia, a vertical deviation

ORBIS

**Figure:**
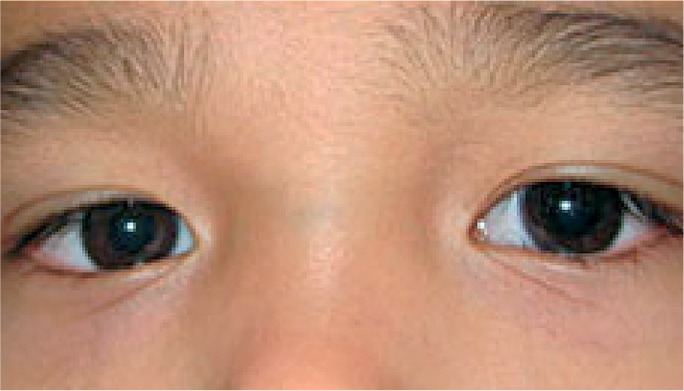
Figure 4. Straight eyes. The light reflex is seen in the centre of the pupils in both eyes. Although this boy's eyes tend to look crossed (because he is looking slightly to the left and the bridge of his nose is broad), the light reflexes in the centre of his pupils confirm that his eyes are straight and aligned.

ORBIS

## Types of strabismus

1. One eye can be deviated inwards (sometimes referred to as being ‘crosseyed’). This is called esotropia.
2. One eye can be deviated outward (sometimes referred to as a ‘wall eye’). This is called exotropia.
3. One eye can be deviated vertically, either upwards (hypertropia) or downwards (hypotropia).

## How does strabismus affect vision?

In a person with strabismus, the eyes are looking at different things; therefore, each eye is sending a different image to the brain. Depending on the individual, the brain will manage these two images in different ways:

1. Very rarely, the person with squint will see two different objects in the same place. The images appear to merge into one, creating what is called visual confusion.
2. More commonly, the person with acute squint will see two images, or ‘double’. The image from the fixing eye will appear normal, and the image from the deviated eye will usually appear blurred.
3. In longer-standing squint, the second or blurred image, produced by the deviated eye, is ignored or suppressed by the brain and only a single object is seen.

In all of the above, the person will have reduced depth perception.

If a person with squint covers their fixing eye, the deviated eye will usually move into the ‘normal’ position, and look at what the person intends to look at. The image produced by that eye will also now be normal (not blurred), even if it was suppressed before.

Sometimes, the vision in the deviated eye will be permanently reduced, even when the fixing eye is covered. This is called **amblyopia,** or ‘lazy eye’.

Amblyopia develops when the strabismus (and suppression) started at a very early age and the brain has been continually suppressing the image from the deviated eye. As a result of this suppression, the part of the visual cortex responsible for interpreting images coming from that eye doesn't receive the stimulation it needs to develop normally. The loss of vision is therefore due to changes in the brain and will persist even if the deviated eye is normal in every way, except for the misalignment.

## Detecting strabismus

Strabismus can be barely detectable in some people, but most of the time the misalignment of the eyes is obvious and can be seen easily.

It may help to shine a small light, such as a penlight, in the patient's eyes. A patient who has straight, aligned eyes (no strabismus) will have a reflection in the centre of each pupil or nearly so (Figure 4). The person who has strabismus will have the reflection in the centre of the pupil of only one eye (the fixing eye) and the other reflection will be seen over the iris or definitely away from the centre of the pupil (the deviated eye). See Figures 5 and 6. Another way to check for strabismus is to cover the fixing eye - the eye that appears to be looking at the target. This will cause the deviating eye (which is not covered) to move in order to look at, or take up fixation, on the target.

**Figure:**
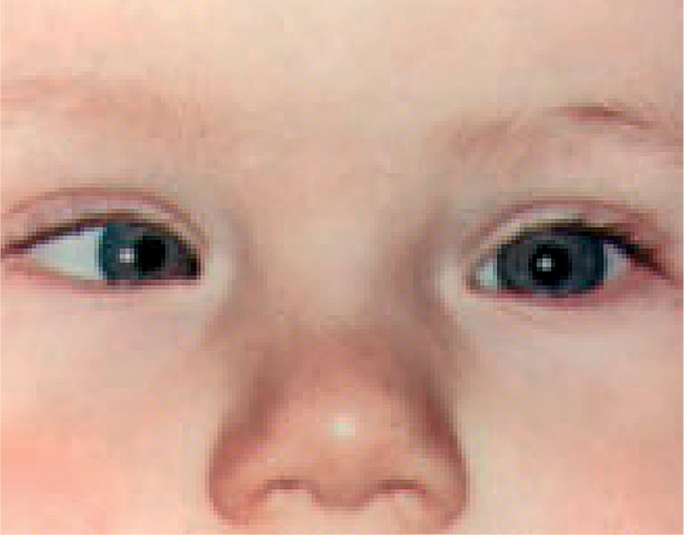
Figure 5. Esotropia in the right eye. The light reflex is central in the left eye (the non-deviated eye), but over the iris in the right eye (the deviated eye).

ORBIS

**Figure:**
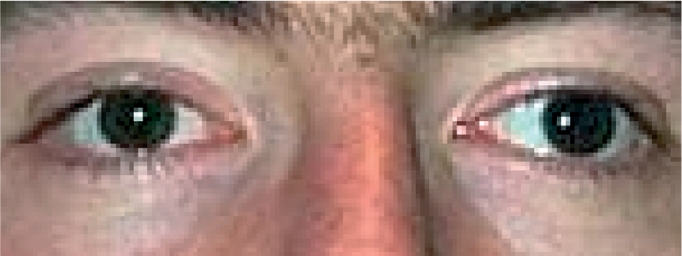
Figure 6. Exotropia in the left eye. The light reflex is central in the right eye (the non-deviated eye), but over the iris in the left eye (the deviated eye).

ORBIS

For example, if the right (fixing) eye of the person in Figure 6 is covered, the left (deviating) eye will move inwards, or toward the nose, confirming the presence of strabismus.

Some individuals with strabismus will have straight eyes part of the time, but will have restriction of movement of one or both eyes, causing strabismus when they look in particular directions. This is called intermittent strabismus.

When checking a person for strabismus, it is therefore necessary to confirm that the eyes can move freely in all directions. There are nine possible positions of gaze, as shown in Figure 7. Check eye movement by holding the patient's head still and asking him or her to follow your finger or a light as you move it to each position.

**Figure:**
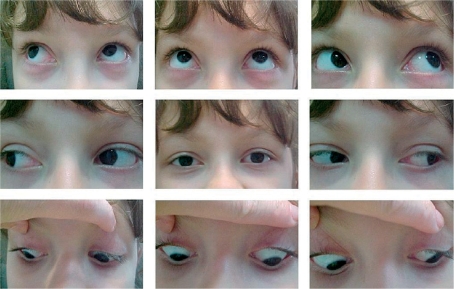
Figure 7. This patient demonstrates the full range of movement of the eyes while maintaining alignment (straight eyes)

ORBIS

The strabismus can be present all of the time or only some of the time. Constant strabismus is more serious. In some instances, the person with constant strabismus assumes an abnormal position of the head to try to keep the eyes aligned.

For example, the child or adult will turn their head or raise or lower the chin to help the eyes to become aligned with what they are looking at (Figure 8). This abnormal head posture can be uncomfortable. If it occurs in a very young child and is persistent, it can cause abnormal growth of the bones of the head.

**Figure:**
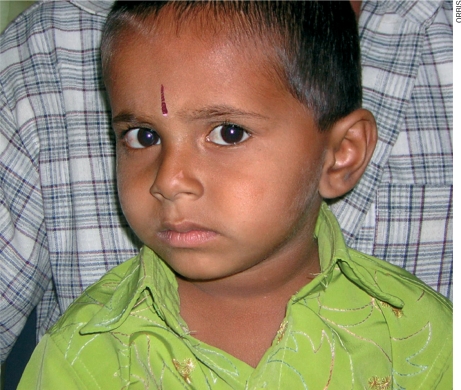
Figure 8. This child assumes an abnormal head posture, facing right while the eyes look to the left, to enable the eyes to be aligned.

ORBIS

## When do patients develop strabismus?

- Some children are born with a tendency for the eyes to cross in (esotropia). This condition tends to run in families and is usually noticed in the first year of life.
- Some strabismus develops later due to defective nerves or muscles, or as a result of trauma.
- Some children develop esotropia when they are aged three to six years old because they are hypermetropic (farsighted) and need spectacles, both to help them focus and to keep their eyes aligned.
- Sometimes strabismus develops due to a serious disease affecting the nervous system or eye. For example, squint (both exotropia and esotropia) can be the first sign of retinoblastoma, a cancer of the eye that is fatal unless treated promptly. Outward deviation of the eye with drooping of the lid occurs due to nerve damage which can be a sign of a brain tumour (Figure 9). Although relatively rare, these causes must be ruled out before treatment for the misalignment can begin.

**Figure:**
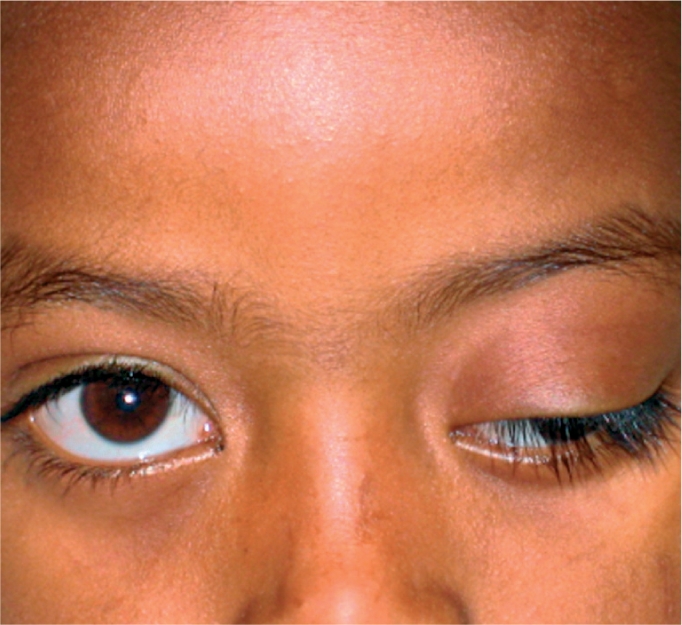
Figure 9. This child has outward deviation of the eye with ptosis, or a drooping lid. This can be the sign of serious neurologic disease, including a brain tumour.

ORBIS

## Managing the patient with strabismus

Regardless of the type of strabismus detected, it is necessary for the affected person, whether child or adult, to have a thorough examination by an ophthalmologist or the best-trained person available.

Once underlying causes such as retinoblastoma have been ruled out, is it important to check the patient for amblyopia **urgently,** as this has to be treated first. The patient's strabismus can then be treated.

It is important to talk to the patient, and the parents, about the treatment plan and what to expect.

### Treating amblyopia

Amblyopia may be reversible if treated as early in life as possible, when the brain and nervous system are still capable of change. If left too late, the amblyopia will be permanent.

Amblyopia is treated by forcing the brain to use the eye with reduced vision (the deviating eye). This is done by covering or handicapping the ‘good’ eye (the fixing eye) with a patch or using medicine to blur vision in that eye. This is usually done from several hours a day to all, or nearly all, of the waking hours and can continue for weeks or months.

By forcing the brain to use the deviated eye, the visual cortex responsible for that eye receives additional visual stimulation which allows it to re-establish, or develop for the first time, a normal level of vision.

Amblyopia treatment should be monitored by an ophthalmologist or orthoptist and the schedule adjusted according to the change in vision.

Urgent treatment of amblyopia in young children is very important. If good vision is restored by patching, and if this good vision is maintained beyond the ages of six to eight years, the child will have a chance to retain good vision in that eye for life.

It is important to note that some children with amblyopia may not have strabismus. Their amblyopia is usually a result of significant differences in refractive error between the two eyes. Because the eyes appear straight, these children can only be identified after careful screening. For them, amblyopia treatment begins by providing glasses that equalise the focus in the two eyes.

It is important to be diligent in finding all children at risk of amblyopia and to convince both the parents and the child of the importance of treatment by patching.

### Treating strabismus

When strabismus is encountered, the first step is to find out the cause. As was stated earlier, this is best done by a doctor who is familiar with the diagnosis and treatment of strabismus in all of its forms.

After taking a careful history, the misalignment is measured and the range of eye movement is checked. After this, refraction under cycloplegia (paralysis of the ciliary muscle of the eye) should be done. Cycloplegia is required so that the full extent of any hypermetropia (farsightedness) can be assessed.

In some children, simply prescribing hypermetropic, or plus, spectacle correction will straighten the eyes. A few children may need bifocals to make sure their eyes stay straight when they are looking at near objects and, in rare cases, to make up for a congenital absence of accommodation (near focusing power).

Other children with strabismus require surgery on the eye muscles to straighten the eyes. This procedure is done in hospital, either as an outpatient or with a short stay. Children require a general anaesthetic.

The surgeon may operate on muscles in one or both eyes: either strengthening the action of the muscles (usually by shortening them), or weakening the action of the muscles (usually by altering where they attach onto the sclera).

After surgery, the patient is expected to have minimal discomfort. Both antibiotic and steroid eye drops or ointment are used for a few days. A patch may also be used, but it is not required in all cases. Patients can return to full activities after a few days, but should not submerge their heads in water for a week or two.

It is important to explain to patients (if old enough) and their parents that a second operation is sometimes needed soon after the first. It is also common for additional surgery to be required if strabismus recurs years after initially successful surgery. For children, alignment attained and maintained to the mid-teens tends to remain stable.

## How would a patient benefit from treatment, optical or surgical, to straighten the eyes?

The most apparent benefits of strabismus treatment are:

1. development or restoration of binocular vision (binocular depth perception)
2. elimination of double vision
3. restoration of normal head posture
4. increased visual field in patients with esotropia
5. creation of a normal appearance.

Even if it is not possible to improve vision, successful strabismus treatment helps the eyes look ‘normal’. This has significant psychological and social benefits, as both children and adults prefer to look like their peers.

## Further study

Eye health care workers, especially those who deal with children, should have a good understanding of the importance of early detection and prompt effective treatment for children with strabismus. More information about strabismus is available on line at **www.cybersight.org** - see Useful Resources on page 11.

